# Supplementary material for: Mouse Breast Carcinoma Monocytic/Macrophagic Myeloid-Derived Suppressor Cell Infiltration as a Consequence of Endothelial Dysfunction in Shb-Deficient Endothelial Cells Increases Tumor Lung Metastasis
Source: Int J Mol Sci. 2021 Oct 25;22(21):11478. doi: 10.3390/ijms222111478 (PMC8583852; doi:10.3390/ijms222111478)
Supplement: Supplementary file 1 [file ijms-22-11478-s001.zip › ijms-1378541-supplementary.pdf]

Supplemental data

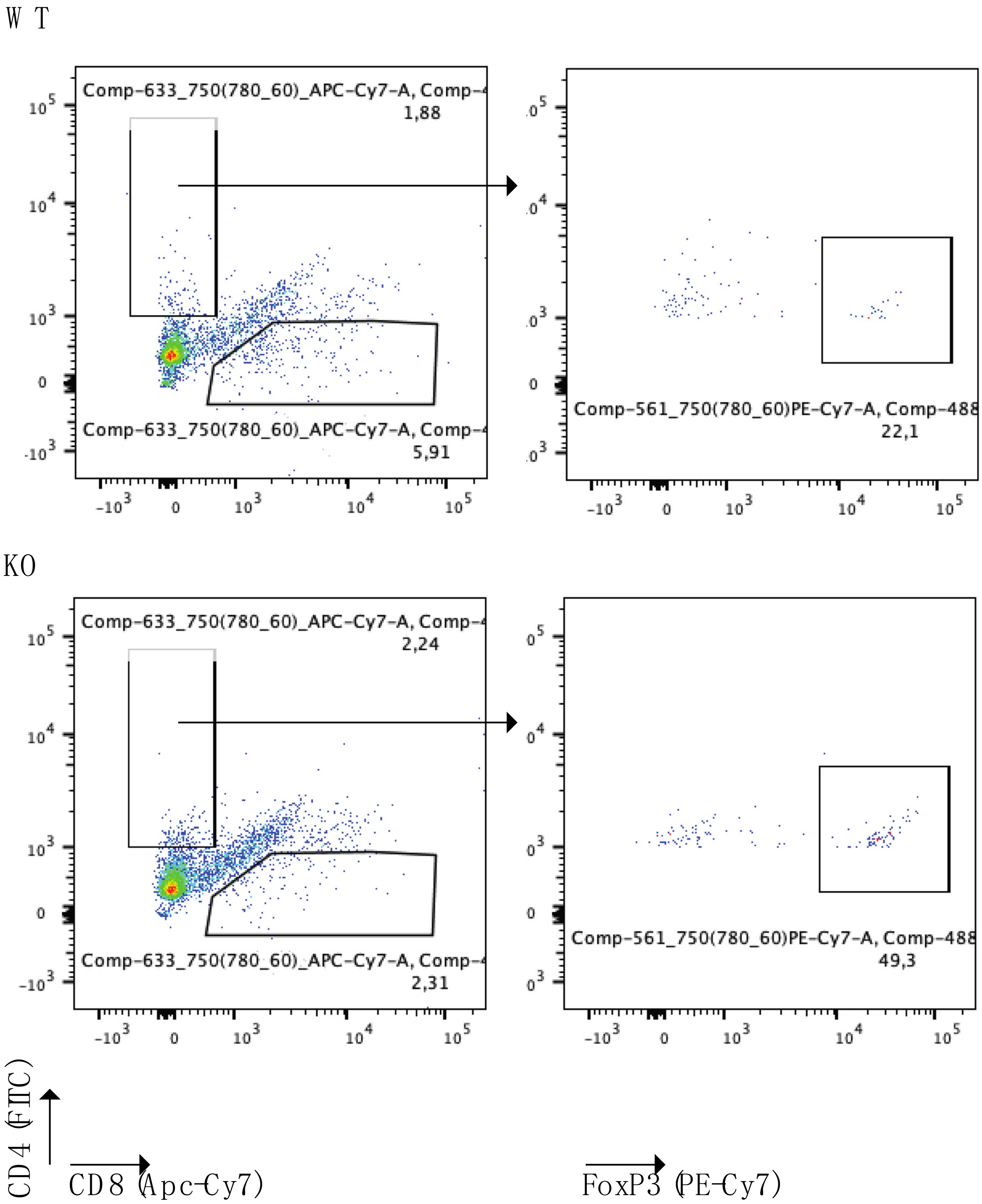

Figure S1: Gating strategy for T cells.

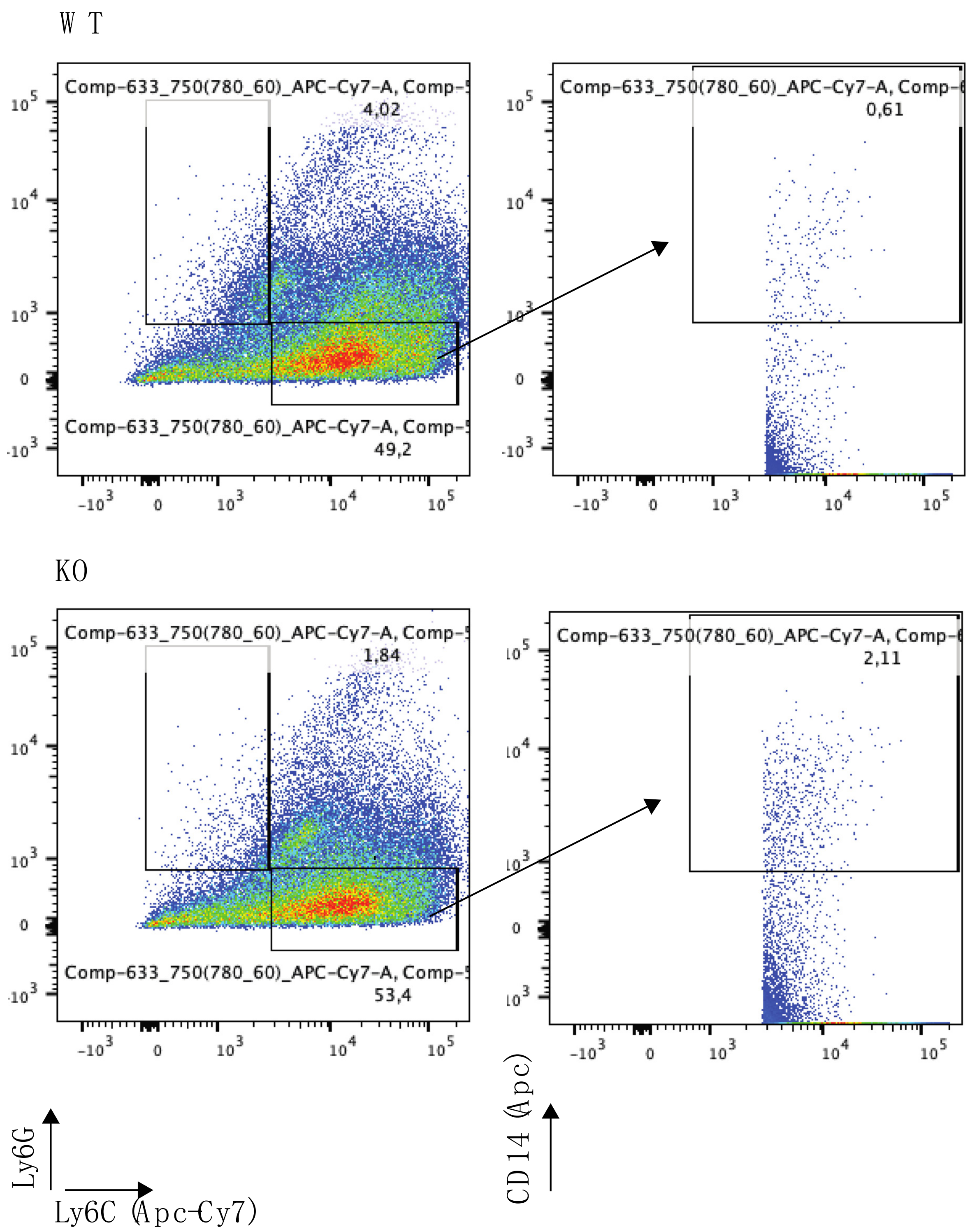

Figure S2: Gating strategy for mMDSC.

Table S1: Immunological reagents.

Antibody target	Host	Supplier	Catalog number	Fluorophore/Tag
Mouse CD31	Rat	BD bioscience	553370	none
Desmin	Goat	Abcam	ab80503	none
Mouse fibrinogen	Goat	Gentaur Mol	GAM/fbg/7s	none
Mouse CD15	Mouse	Biolegend	125604	Biotin
Rat IgG	Donkey	Invitrogen	A21209	Alexa 594
Goat IgG	Donkey	Invitrogen	A11055	Alexa 488
Mouse CD4	Rat	eBioscience	11-0041-82	FITC
Mouse CD8a	Rat	BD bioscience	560182	APC-Cy7
FoxP3	Rat	eBioscience	25-5773-82	PE-Cy7
Mouse CD11b	Hamster	Biolegend	101206	FITC
Mouse CD11c	Hamster	Biolegend	117310	APC
Mouse Ly6C	Rat	Biolegend	128026	APC-Cy7
Mouse Ly6G	Rat	Biolegend	127608	PE
Mouse CD14	Rat	Biolegend	123312	APC
Streptavidin		Biolegend	405214	PercP-Cy5.5
**MACS microbeads from Miltenyi Biotech**				
Anti-Biotin	130-090-485			
Anti-CD31	130-097-418			
Anti-CD11b	130-049-601			

Table S2: qPCR primer sequences.

Gene	Forward primer	Reverse primer
Actb	CAC TAT TTG GCA ACG AGC GG	TCC ATA CCC AAG AAG GAA GGC
Shb	TTT GAT GCC AAG AGC GAC CT	GAG TCT GAG TCC ACG CTC TG
Il6	CTTCCATCCAGTTGCCTTCTTG	AATTAAGCCTCCGACTTGTGAAG
Il1b	ACGGATTCCATGGTGAAGTC	GAGTGTGGATCCCAAGCAAT
Il17a	TCTTTAACTCCCTTGGCGCA	TCAGGGTCTTCATTGCGGTG
Tnf	ACTGCCAGAAGAGGCACTCC	CGA TCA CCC CGA AGT TCA
Ifng	CCT GGG GCC TAG CTC TGA	CAG CCA GGA ACA GCC ATG AG
Tbx21	CCACCTGTTGTGGTCCAAGT	TAATGGCTTGTGGGCTCCAG
Cxcl1	ACTCAAGAATGGTCGCGAGG	ACTTGGGGACACCTTTTAGCA
Cxcl9	CCAAGCCCCAATTGCAACAA	AGTCCGGATCTAGGCAGGTT
Cxcl10	GCT GCC GTC ATT TTC TGC	TCT CAC TGG CCC GTC ATC
Cxcl11	CGGCTGCGACAAAGTTGAAG	TATGAGGCGAGCTTGCTTGG
Cxcl12	TGCATCAGTGACGGTAAACCA	TTCTTCAGCCGTGCAACAATC
Cxcr4	ATG ACC AGG ATC ACC AAT CCA	CGG GAT GAA AAC GTC CAT TT
Ccl2	CACTCACCTGCTGCTACTAC	GCTTGGTGACAAAAACTACAGC
Ccl3	GCAACCAAGTCTTCTCAGCG	GGAAAATGACACCTGGCTGG
Ccl4	AACCTAACCCCGAGCAACAC	TGCCGGGAGGTGTAAGAGAA
Ccl20	ATCTGTGTGCGCTGATCCAA	CTCCTTGGGCTGTGTCCAAT
Ccl21	GTGATGGAGGGGGTCAGG	GGGATGGGACAGCCTAAA
Ccl22	TCT GAT GCA GGT CCC TAT GGT	TTA TGG AGT AGC TTC TTC AC
Ccr7	TGTACGAGTCGGTGTGCTTC	GGTAGGTATCCGTCATGGTCTTG
Csf1	AACAGCTGCTTCACCAAGGA	GCTGGAGAGGAGTCTCATGG
Csf2	GGCCTTGGAAGCATGTAGAGG	GGAGAACTCGTTAGAGACGACTT
Csf3	ATTGGCTCAACTTTCTGCCCAG	CTGACAGTGACCAGGGGAAC
Nos2	GTT CTC AGC CCA ACA ATA CAA GA	GTG GAC GGG TCG ATG TCA C
Gata3	CGA GAT GGT ACC GGG CAC TA	GAC AGT TCG CGC AGG ATG T
Arg1	TGG CTT GCG AGA CGT AGA C	GCT CAG GTG AAT CGG CCT TTT
Il4	CGG AGA TGG ATG TGC CAA AA	GCA CCT TGG AAG CCC TAC AG
Il5	CTCTGTTGACAAGCAATGAGACG	TCTTCAGTATGTCTAGCCCCTG
Il13	CAG CAT GGT ATG GAG TGT GG	CCT CTT GCC GAC ATG GAA TA
Il10	CTG GAC AAC ATA CTG CTA ACC G	GGG CAT CAC TTC TAC CAG GTA A
Il12a	AGCCTTCCTCCTATCAGCCA	GTGTGCAGCCCCTAGAATGT
Cd4	GAGGGGTGAACCAGACAGTG	ATTCGTGCTGCCTGGCG
Cd8a	CTAGACGTGGAGGAAGACGC	GAGGACCATGGGTGACTCTT
Foxp3	TTA TCC GAT GGG CCA TCC T	CAA AGC ACT TGT GCA GGC TC
Gzmb	CTGCAAAAACAGCTCTGTCCAA	CTCCTCCTTAGCCGTGATGT
S100a9	TGGGCTTACACTGCTCTTACC	GGTTATGCTGCGCTCCATCT
Itgam	CCT TCA TCA ACA CAA CCA GAG TGG	CGA GGT GCT CCT AAA ACC AAG C
Itgax	CCT GAG GGT GGG CTG GAT	GCC AAT TTC CTC CGG ACA T
Adgre1	GGA AAC CAC CAT GTT AGC TGC	GGA AAC CAC CAT GTT AGC TGC
Pdcd1	ATCTACCTCTGTGGGGCCAT	AGGTCTCCAGGATTCTCTCTGT
Cd274	GGCAGGAGAGGAGGACCTTA	CTGTGATCTGAAGGGCAGCA
Ctla4	ACGCAGATTTATGTCATTGATCCAG	AACCCCAAGCTAACTGCGAC
Tgfb1	CAGCCCTGCTCACCGTCGTG	GGTTTGTGGCTCCCGAGGGC
Tgfb2	CACACACACACACACACACACC	TGCAAAGCTCGCCAACCATCTC
Tgfb3	AAAGGATCACCACAACCCACAC	TCTTCTTCCTCTGACTGCCCTG
